# Incidence of Clinician-Diagnosed Lyme Disease, United States, 2005–2010

**DOI:** 10.3201/eid2109.150417

**Published:** 2015-09

**Authors:** Christina A. Nelson, Shubhayu Saha, Kiersten J. Kugeler, Mark J. Delorey, Manjunath B. Shankar, Alison F. Hinckley, Paul S. Mead

**Affiliations:** Centers for Disease Control and Prevention, Fort Collins, Colorado, USA (C.A. Nelson, K.J. Kugeler, M.J. Delorey, A.F. Hinckley, P.S. Mead);; Centers for Disease Control and Prevention, Atlanta, Georgia, USA (S. Saha, M.B. Shankar)

**Keywords:** Lyme disease, Borrelia burgdorferi, surveillance, United States, vector-borne infections, zoonoses, Ixodes, Emerging Infections Program, EIP

## Abstract

National surveillance provides important information about Lyme disease (LD) but is subject to underreporting and variations in practice. Information is limited about the national epidemiology of LD from other sources. Retrospective analysis of a nationwide health insurance claims database identified patients from 2005–2010 with clinician-diagnosed LD using International Classification of Diseases, Ninth Revision, Clinical Modification, codes and antimicrobial drug prescriptions. Of 103,647,966 person-years, 985 inpatient admissions and 44,445 outpatient LD diagnoses were identified. Epidemiologic patterns were similar to US surveillance data overall. Outpatient incidence was highest among boys 5–9 years of age and persons of both sexes 60–64 years of age. On the basis of extrapolation to the US population and application of correction factors for coding, we estimate that annual incidence is 106.6 cases/100,000 persons and that ≈329,000 (95% credible interval 296,000–376,000) LD cases occur annually. LD is a major US public health problem that causes substantial use of health care resources.

## Abstract 2

Extrapolation from a large medical claims database suggests that 329,000 cases occur annually.

Lyme disease (LD) is a zoonotic infection transmitted by *Ixodes* spp. ticks and caused by the spirochete *Borrelia burgdorferi*. Signs and symptoms of infection range in severity and can include erythema migrans, arthritis, facial palsy, radiculoneuropathy, arrhythmia, and meningitis. Most patients recover fully after antimicrobial treatment (*1*,*2*); however, serious illness and even deaths have been reported, although rarely (*3*–*5*). In the United States, LD is the fifth most commonly reported nationally notifiable disease; ≈36,000 confirmed and probable cases were reported in 2013 (*6*). US cases are concentrated heavily in the Northeast and upper Midwest (*7*).

Surveillance for LD in the United States is based on reports submitted by laboratories and health care providers to state and local health departments. These reports provide valuable insight into the age and sex distribution of patients with LD and the seasonality and geographic distribution of cases, and they enable monitoring of disease trends over time. Unfortunately, underreporting and variation in surveillance practices limit the ability of routine surveillance to capture the true overall frequency of LD within the population (*8*). Studies conducted during the 1990s in high-incidence states suggest that LD cases are underreported by a factor of 3 to 12 (*9*–*12*). These studies were limited to specific states and do not necessarily reflect underreporting nationwide.

Medical claims data provide an additional source of information about the epidemiology and public health importance of LD. Because these data are based on billing records submitted by clinicians for reimbursement, they are less prone to underreporting than are routine surveillance data that require additional documentation. We used information from a large, nationwide medical claims database to 1) describe the epidemiology of LD diagnosed by clinicians, 2) identify similarities and differences with surveillance data, and 3) estimate the number of LD cases per year in the United States.

## Methods

### Medical Claims Database

During 2013–2014, we retrospectively analyzed the 2005–2010 Truven Health MarketScan Commercial Claims and Encounters Database, which contains health insurance claims information for a median of 27 million persons each year. The database contains records for persons 0–64 years of age with employer-provided health insurance and includes information about employees and their spouses and dependents from all 50 states. Deidentified data on enrollee demographics, outpatient and emergency department visits, inpatient admissions, and prescription drugs are included.

Each patient encounter record is assigned >1 diagnostic code from the International Classification of Diseases, Ninth Revision, Clinical Modification (ICD-9-CM), by a clinician or billing specialist. Inpatient admissions in the database include 1 principal diagnosis and up to 14 secondary diagnoses. Outpatient encounters include up to 4 associated ICD-9-CM codes but do not distinguish between principal and secondary diagnoses. Medication information is available for most enrollees for prescription drugs filled at outpatient pharmacies.

### Epidemiology of Clinician-Diagnosed LD in the MarketScan Database

The study population comprised persons enrolled in a participating health plan for the entirety of any year during 2005–2010 and for whom prescription drug information was available. For this analysis, we defined an inpatient event as a hospital admission with the ICD-9-CM code for LD (088.81) as the principal diagnosis or the 088.81 code as a secondary diagnosis plus a principal diagnosis consistent with an established manifestation of LD or plausible co-infection (Technical Appendix).

We defined an outpatient event as any outpatient or emergency department visit with the 088.81 code plus a prescription filled for an antimicrobial drug recommended by the Infectious Diseases Society of America for LD treatment (*13*). Three additional antimicrobial drugs also were included because they were closely related to a recommended antimicrobial drug or were a known historical treatment that some practitioners might still prescribe (Technical Appendix). Only prescriptions of at least 7 days’ duration and filled ±30 days from the visit date were considered.

The first outpatient or inpatient event of each year that met the study definition was considered the incident diagnosis for a patient. The date of admission or first outpatient visit that met study inclusion criteria was considered the date of the event. A separate LD diagnosis that met inclusion criteria at least 1 year after the previous diagnosis was included as a new incident event. When both an outpatient event and inpatient admission occurred within 1 year, only the inpatient admission was considered. To maintain consistency with US surveillance data, location was based on the patient’s county of residence, not where care was provided.

### National Surveillance and US Population Data

State and local health officials report LD cases to the Centers for Disease Control and Prevention (CDC) through the National Notifiable Diseases Surveillance System according to standardized case definitions (*14*). For comparison with MarketScan findings, we analyzed surveillance cases reported during 2005–2010. Cases reported during 2005–2007 reflected a surveillance case definition comprising confirmed cases only. Beginning in 2008, a revised case definition was in place that altered the laboratory criteria and distinguished between confirmed and probable cases; cases reported during 2008–2010 included both categories (*15*). US Census 2010 population data were used for population comparisons and extrapolations (*16*).

### Estimation of the Number of Clinician-Diagnosed LD Cases

To estimate the total number of patients with clinician-diagnosed LD in the United States, we calculated age- and county-specific rates derived from the MarketScan database and applied them to the 2010 population of each corresponding county. Counts for all US counties were then summed. Because the MarketScan database is limited to persons <65 years of age, these calculations do not include clinician-diagnosed cases among persons >65 years. To adjust for this exclusion, we multiplied by a correction factor of 1.17. This correction factor was inferred from the age distribution of LD patients reported through national surveillance. During 2005–2010, persons <65 years of age accounted for 85.8% of LD cases reported through national surveillance. Therefore, we multiplied the estimated number of cases among persons <65 years by 1.00/0.858, or 1.17, to arrive at an estimate of cases in all age groups.

The estimated number of patients with clinician-diagnosed LD was based on extraction of a single ICD-9-CM code. Research has shown, however, that clinician diagnosis of a medical condition does not necessarily correlate with existence of the ICD-9-CM code in the chart (*17*,*18*). The primary reasons are coding errors and inclusion of codes for accompanying symptoms but not the specific disease (e.g., coding for joint pain but not LD) (*17*,*19*). To correct for omission of the 088.81 code, we relied on 4 evaluations of coding patterns for patients in whom LD was diagnosed. The Minnesota Department of Health found the 088.81 code was present in 145 (56.4%) of 257 charts for which a clinician documented a new case of LD (E. Schiffman, pers. comm.). A Maryland Department of Health and Mental Hygiene study found the 088.81 code in 45 (44.6%) of 101 charts from patients in whom LD was diagnosed and reported by clinicians or clinical centers (*20*). Furthermore, the New York State Department of Health found the 088.81 code in 114 (41.8%) of 273 charts from patients in whom LD was diagnosed (J. White, pers. comm.). Finally, the Tennessee Department of Health found the 088.81 code listed at least once in 9 (37.5%) of 24 charts from patients with Blue Cross Blue Shield insurance in whom LD was diagnosed and who were reported to the Department of Health (*21*). Thus, of 655 collective charts from LD patients, 313 charts had 088.81. Therefore, to account for patients in whom LD was diagnosed but whose charts were not coded with 088.81, we multiplied the estimated number of cases with 088.81 by a correction factor calculated as follows: 313/655 = 1/*x*, where *x* = 2.09.

### Statistical Methods

We calculated direct standardization and descriptive statistics using SAS software version 9.3 (SAS Institute, Cary, NC, USA). The χ^2^ test was used to compare categorical data. Cramer’s V values were calculated to compare distributions by using R statistical software version 3.1.1 (http://www.r-project.org/). Methods for credible interval calculation are provided in the online Technical Appendix.

### Ethics Review

CDC human subjects review of the protocol determined it was not research involving human subjects. Thus, Institutional Review Board approval was not required.

## Results

### Study Population

The final study dataset comprised 103,647,966 person-years of observation (median 17,309,054 persons/year). Median age of the study population was 37.0 years; 51.9% of patients were female. For comparison, the median age of the US population in 2010 was 37.2 years, and 50.8% of the population was female.

### Epidemiology of Clinician-Diagnosed LD and Comparisons with Surveillance Data

**Figure 1:**
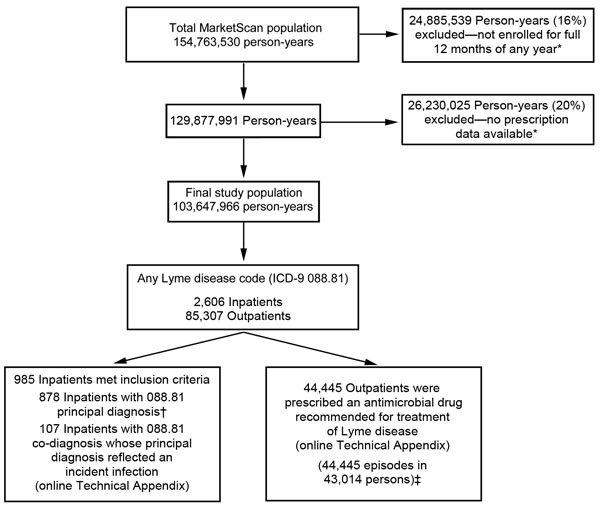
Study population and number of patients with clinician-diagnosed Lyme disease in the MarketScan database, United States, 2005–2010. *Persons not enrolled for the full 12 months of any year and who did not have prescription data were removed from both the numerator and denominator for rate calculations. Therefore, removal of these persons did not substantially affect rate calculations and the final estimated number of cases. †One repeat inpatient was excluded (admitted in a subsequent year but <365 days after initial admission). No repeat admissions occurred >365 days after initial admission. ‡A total of 2,945 repeat outpatients (seen in a subsequent year but <365 days after previous year’s visit) were excluded (Technical Appendix). ICD–9, International Classification of Diseases, Ninth Revision.

**Figure 2:**
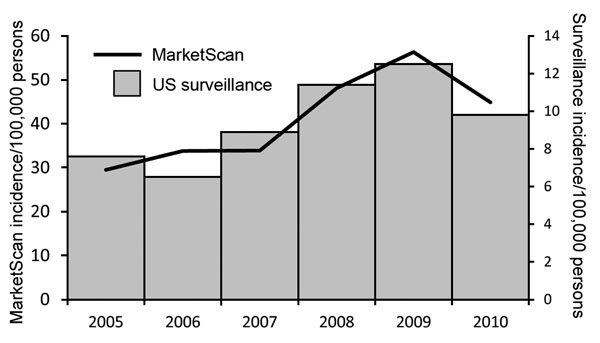
Trends of annual incidence of Lyme disease in MarketScan compared with trends in incidence from US surveillance, 2005–2010. Incidence is per 100,000 persons. Trends in interannual incidence fluctuation did not differ significantly between MarketScan and US surveillance (χ^2^ test, p = 0.81). *Cases reported through the National Notifiable Diseases Surveillance System. During 2005–2007, incidence was calculated as the number of confirmed cases/100,000 persons; during 2008–2010, incidence was calculated as the number of confirmed and probable cases/100,000 persons. US 2010 Census population estimates were used as the denominator for surveillance data incidence calculations.

A total of 45,430 clinician-diagnosed LD events were identified during 2005–2010; 985 (2.2%) were inpatient admissions and 44,445 (97.8%) were outpatient events (Figure 1). Average annual incidence within the MarketScan population was 44.8 events per 100,000 persons, with a peak of 56.3 events per 100,000 persons in 2009 (Figure 2). Interannual fluctuation in incidence in MarketScan data was similar to that in surveillance data (χ^2^ test, p = 0.81; Cramer’s V = 0.037).

**Figure 3:**
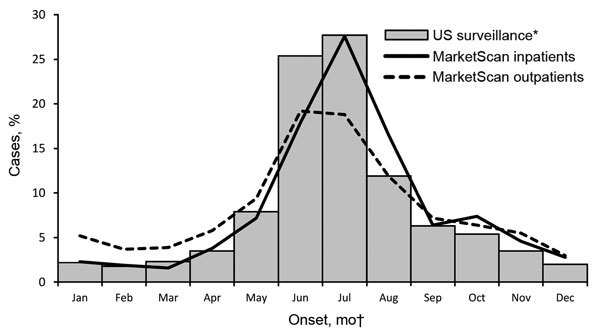
Seasonal distribution of inpatient and outpatient clinician-diagnosed Lyme disease in MarketScan compared with US surveillance cases, 2005–2010. *Because information about hospitalization is not consistently captured by surveillance, US surveillance data include both inpatients and outpatients. †Date of symptom onset for surveillance cases; date of admission or first outpatient visit for MarketScan events.

Clinician-diagnosed LD events peaked during the summer months, although more so for inpatient admissions (61.9% occurred during June–August) than for outpatient events (50.0% occurred during June–August). In comparison, 65.0% of cases reported through surveillance occurred during June–August (Figure 3). Seasonal distribution of LD events in MarketScan differed significantly from cases reported through surveillance, though this is likely an artifact of the large sample sizes since the magnitude of Cramer's V suggests little difference in the distributions (inpatients: χ^2^ test, p<0.001, Cramer’s V = 0.019; outpatients: χ^2^ test, p<0.001, Cramer’s V = 0.154).

**Figure 4:**
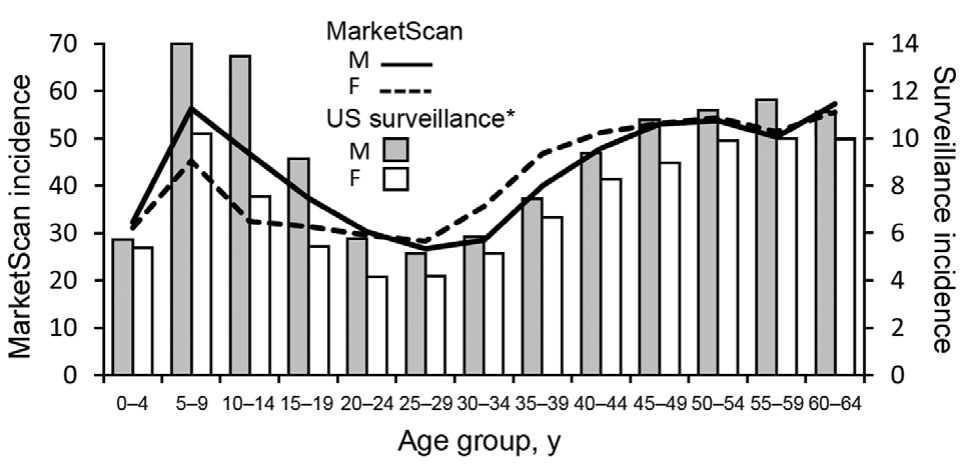
Comparison of trends in the age and sex distribution of persons with Lyme disease in MarketScan with US surveillance, 2005–2010. Incidence is per 100,000 persons. Age distribution of persons with Lyme disease in MarketScan did not differ from those reported through US surveillance (male patients: χ^2^ test, p = 0.57; female patients: χ^2^ test, p = 0.43). *US 2010 Census population estimates were used as the denominator for surveillance incidence calculations.

Age distribution for both male and female patients did not differ significantly from the distributions reported through surveillance (male: χ^2^ test, p = 0.57, Cramer’s V = 0.054; female: χ^2^ test, p = 0.43, Cramer’s V = 0.054) (Figure 4). For inpatients, the highest average annual admission rates were for boys 5–9 years of age (1.8 admissions/100,000 persons) and men 60–64 years of age (1.9 admissions/100,000 persons). For outpatient events, the highest annual incidences were for boys 5–9 years of age (54.5 events/100,000 persons), men 60–64 years of age (55.4 events/100,000 persons), and women 60–64 years of age (54.7 events/100,000 persons). Relative to surveillance data, the incidence of clinician-diagnosed LD was higher than expected for women 15–34 years of age.

**Figure 5:**
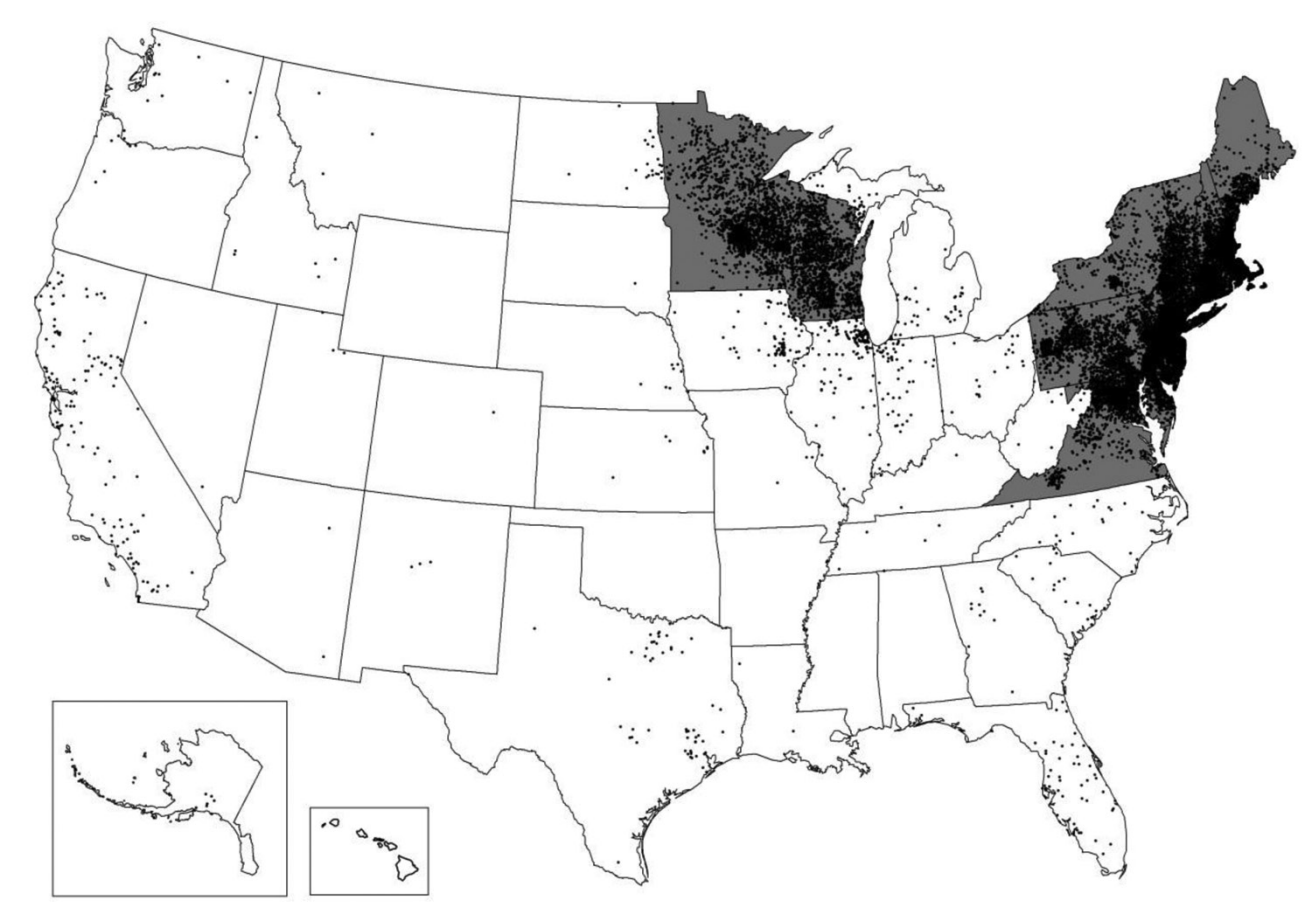
Comparison of states and district with highest incidence per 100,000 persons of Lyme disease in MarketScan (gray fill) and US surveillance (black dots), 2005–2010. Each dot is placed randomly within the county of residence for each confirmed Lyme disease case reported through surveillance during 2010.

The 15 states and district with the highest average incidence represented 80.6% of clinician-diagnosed LD and were as follows, in descending order: Connecticut, Rhode Island, Maryland, New Jersey, Massachusetts, New York, New Hampshire, Pennsylvania, Maine, Delaware, Virginia, Vermont, Wisconsin, District of Columbia, and Minnesota (Figure 5). These same 15 states and district were seen in surveillance data, although the rank order differed slightly, and they constituted a significantly greater proportion (96.3%) of reported cases (χ^2^ test, p<0.001).

### Estimated Number of Clinician-Diagnosed LD Cases

Direct standardization of clinician-diagnosed LD and addition of estimated cases in persons >65 years of age produced an estimate of 157,137 cases per year, which was multiplied by 2.09 to correct for omission of the 088.81 code in patient charts. This calculation yielded a national estimate of 329,000 LD cases per year during 2005–2010 (95% credible interval 296,000–376,000). On the basis of this number, the estimated incidence of clinician-diagnosed LD in the United States during this period was 106.6 cases per 100,000 persons per year. In comparison, average US incidence according to surveillance data during this period was 9.4 cases per 100,000 persons per year.

Sensitivity analyses showed that the correction factor for patients in whom LD was diagnosed but who were not given the 088.81 code had the greatest influence on the final estimate (Technical Appendix). For example, a 10% increase in this correction factor led to a 6% increase in the final estimate, and a 30% decrease led to a 12% decrease in the final estimate.

## Discussion

Using medical claims data, we estimated that 329,000 (95% credible interval 296,000–376,000) LD cases occur annually in the United States, which emphasizes the substantial public health effect of this disease. This estimate is consistent with findings from a recent study of diagnostic laboratories that yielded an estimate of 288,000 (range 240,000–444,000) infections among patients for whom a laboratory specimen was submitted in 2008 (*22*). As expected, our estimate is slightly higher because it also includes LD cases diagnosed without laboratory testing (i.e., clinical diagnosis based on presence of erythema migrans after exposure in a Lyme-endemic area).

Presence of a diagnostic code in the chart or a clinician diagnosis of an infectious condition does not necessarily signify a true infection (*19*). Possible reasons include rule-out diagnoses, codes for medical history but not incident infections, and overdiagnosis (incorrect diagnosis of LD when the patient has a different condition). Rule-out diagnoses and medical history codes most likely were reduced—but not completely eliminated—by including only outpatients treated with an antimicrobial drug recommended for LD. Overdiagnosis of LD is not uncommon given that, in some circumstances, the differential diagnosis for symptoms of LD can be broad (*23*–*25*). Studies of patient charts with the 088.81 code found that 37.9% in Maryland and 55.2% in Wisconsin were classified after chart review as noncases according to the surveillance case definition (*12*,*20*). Thus, we cannot exclude the possibility that some of the ≈329,000 patients in whom LD was diagnosed were not infected with *B. burgdorferi*.

Epidemiologic patterns of clinician-diagnosed LD were similar to patterns among cases reported through national surveillance; for example, incidence was highest among boys 5–9 years of age and persons 60–64 years of age of both sexes, which is believed to be attributable partially to behavioral factors and increased exposure to ticks in these age groups. However, some discrepancies were also noted. Specifically, incidence of clinician-diagnosed LD was higher than expected among women 15–44 years of age. A study of records with the 088.81 code using Maine’s statewide electronic database of inpatient and outpatient encounters also found a higher percentage female patients compared with surveillance data (*26*). This finding might be attributable to differential overdiagnosis of LD in these groups, variations in insurance coverage and health care–seeking behavior, or other factors. Studies in Europe have found sex discrepancies in risk for tick bites and clinical presentation of LD that should be explored further in US research studies (*27*,*28*).

The estimated number of clinician-diagnosed LD cases in the United States is higher than the number reported through routine surveillance and consistent with previous estimates of LD underreporting (*10*,*11*). Underreporting occurs with other notifiable conditions and should not be confused with lack of treatment (*8*). Indeed, our study confirms that many LD cases not formally reported are nevertheless diagnosed and treated by clinicians. Furthermore, underreporting aside, the general concordance in LD epidemiology seen in MarketScan and surveillance data underscores that LD surveillance serves its central purpose: to identify and track patterns of disease.

Primary advantages of this study are the large sample size, ability to circumvent the obstacles and biases inherent in routine reporting mechanisms, detailed information about clinical and prescription data, and ability to follow patient data over time. Unfortunately, use of the 088.81 code to estimate *B. burgdorferi* infections required several assumptions and correction factors. We calculated these correction factors using data from several analyses, each of which has its own inherent limitations and some of which have not yet been published. Nevertheless, the findings from these analyses were generally consistent with each other and with results expected on the basis of public health experience.

Our findings are subject to additional limitations. The MarketScan population is a convenience sample of the US population <65 years of age; although it is overall fairly representative, some differences exist. For example, certain age groups (20- to 29-year-olds) were 2%–3% underrepresented, and others (50- to 59-year-olds) were 2% overrepresented, compared with the US population. Although our calculations adjust for age and geographic differences for all persons <65 years of age, other differences from the general population probably remain. In addition, the MarketScan database does not include military personnel, uninsured persons, or Medicaid/Medicare enrollees for whom risk for LD might differ from that of privately insured persons.

Our study highlights the need for continued coding research, particularly as health departments explore the feasibility of using electronic medical records to facilitate LD reporting. Additional information about LD coding practices will enable robust comparisons of ICD codes related to actual cases and facilitate future research using medical databases. In addition, ongoing research using the MarketScan databases and other sources will elucidate detailed epidemiologic and clinical aspects of LD that are not apparent in standard surveillance data.

In conclusion, our findings underscore that LD is a considerable public health problem, both in terms of number of cases and overall health care use. Furthermore, as with other conditions, underreporting in the national surveillance system remains a challenge. Continued research and education are necessary to enhance prevention efforts and improve diagnostic accuracy to reduce the effects of this disease.

Technical Appendix Codes from the International Classification of Diseases, Ninth Revision, Clinical Modification, for established manifestations of Lyme disease (LD) or plausible co-infections; antimicrobial drugs used for treatment of LD and establishment of inclusion criteria for outpatient events; and sensitivity analysis and calculation of credible interval for LD estimate.
