# Supplementary material for: Incidence of Clinician-Diagnosed Lyme Disease, United States, 2005–2010
Source: Emerg Infect Dis. 2015 Sep;21(9):1625–31. doi: 10.3201/eid2109.150417 (PMC4550147; doi:10.3201/eid2109.150417)
Supplement: Technical Appendix — Codes from the International Classification of Diseases, Ninth Revision, Clinical Modification, for established manifestations of Lyme disease (LD) or plausible co-infections; antimicrobial drugs used for treatment of LD and establishment of inclusion criteria for outpatient events; and sensitivity analysis and calculation of credible interval for LD estimate. [file 15-0417-Techapp-s1.pdf]

Article DOI: http://dx.doi.org/10.3201/eid2109.150417

Incidence of Clinician-Diagnosed Lyme Disease, United States, 2005–2010

Technical Appendix

Codes from the International Classification of Diseases, Ninth Revision, Clinical Modification, for Established Manifestations of Lyme Disease or Plausible Co-infections

Specific Manifestation (code)

Meningitis (320.7)

Meningitis, unspecified (322.9)

Meningitis due to unspecified bacterium (320.9)

Acute pericarditis (420.xx)

Myocarditis (422.xx, 429.xx)

Conduction disorders (426.xx)

Arthropathy (716.9x)

Arthropathy associated with infections (711.xx)

Pain in joint (719.4x)

Joint effusion (719.0x)

Facial weakness (438.83)

Injury to facial nerve (951.4) or other specified cranial nerves (951.8)

Neuralgia, neuritis, and radiculitis unspecified (729.2)

Nerve lesions (353.xx, 354.xx, 355.xx)

Acute infective polyneuritis (357.0)

Polyneuropathy in other diseases classified elsewhere (357.4)

Unspecified inflammatory and toxic neuropathies (357.9)

Co-infection (code)

Babesiosis (088.82)

Anaplasmosis / Ehrlichiosis (082.4x)

Antimicrobial Drugs Used for Treatment of Lyme Disease and Establishment of Inclusion Criteria for Outpatient Events

Amoxicillin

Amoxicillin/clavulanic acid[1]

Azithromycin or azithromycin dihydrate

Doxycycline (all forms)

Cefotaxime sodium

Ceftriaxone sodium

Cefuroxime axetil

Clarithromycin

Erythromycin—all forms except lactobionate (intravenous [IV]), gluceptate (IV), thiocyanate (not available in the United States), and ethylsuccinate/sulfisoxazole

Minocycline hydrochloride[1]

Penicillin G (benzathine, procaine, or potassium)

Tetracycline hydrochloride[1]

[1]These antimicrobial drugs are not formally recommended for treatment of Lyme disease but are closely related to the recommended drug or are a known historical treatment that some practitioners might still prescribe.

Sensitivity Analysis and Calculation of Credible Interval for Lyme Disease Estimate

Sensitivity Analysis

Each correction factor was increased and decreased by factors indicated on the x-axis (e.g., 0.1 corresponds to a ±10% change in the correction factor). The y-axis represents the relative change in our final estimate of annual *Borrelia burgdorferi* infections with each incremental change in the correction factor. The lines above 1.0 represent how much the final Lyme disease (LD) case estimate increases with positive changes in each correction factor (e.g., 1.2 corresponds to a 20% increase in the final estimate). The lines below 1.0 represent how much the LD case estimate decreases with negative changes.

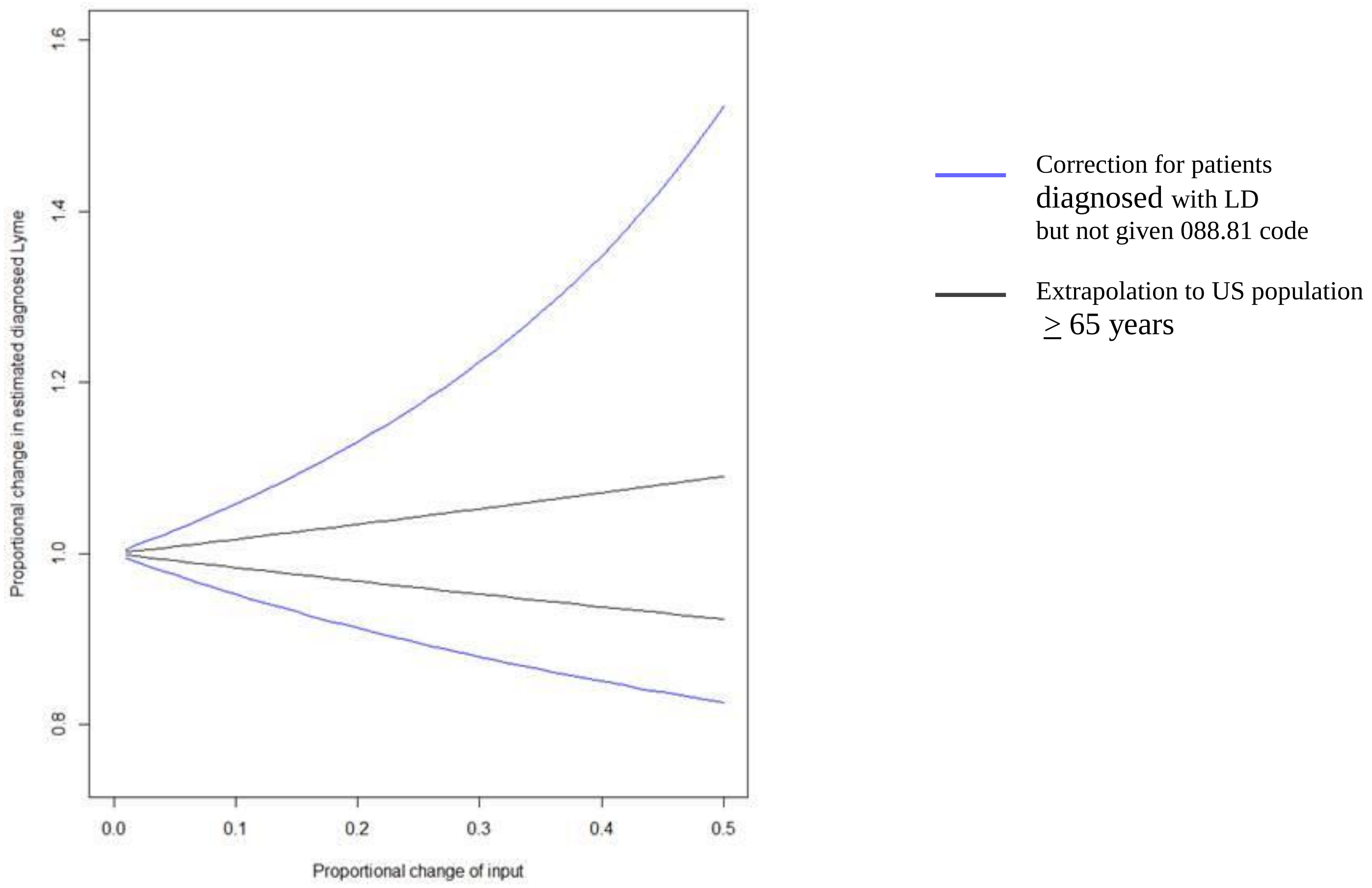

Sensitivity analysis demonstrates that the correction factor for patients in whom LD was diagnosed but who were not given the 088.81 code has the largest effect on the final estimate. (Technical Appendix Table).

Technical Appendix Table. Magnitude of change in the final estimate based on specific changes in each correction factor

	Relative change in final estimate when correction factor is increased or decreased by 0.1/0.3			
**Correction factor**	–0.1	+0.1	–0.3	+0.3
Correction for patients in whom LD was diagnosed but who were not given ICD-9-CM 088.81 code	0.95	1.06	0.88	1.22
Extrapolation to US population ≥65 years of age	0.98	1.02	0.95	1.05

*ICD-9-CM, International Classification of Diseases, Ninth Revision, Clinical Modification; LD, Lyme disease.

Calculation of Credible Interval

Credible intervals were obtained through simulation. Each correction factor was derived from a sample proportion based on the studies described in the methods. These sample proportions were considered binomial random variables because each had a fixed sample size and a fixed probability. Each correction factor, or binomial random variable, was simulated 20,000 times. For each simulation, we computed the values in the flow chart to create a distribution of LD estimates. The 2.5th and 97.5th percentiles from the simulated distribution of LD estimates represent the lower and upper bounds, respectively, for the credible interval.
